# 10 Hz flicker improves recognition memory in older people

**DOI:** 10.1186/1471-2202-7-21

**Published:** 2006-03-05

**Authors:** Jonathan Williams, Deepa Ramaswamy, Abderrahim Oulhaj

**Affiliations:** 1 OPTIMA, Radcliffe Infirmary, Woodstock Road, Oxford, OX2 6HE, UK; 2 Dept. of Psychiatry Unit II, Christian Medical College, Vellore 632002, India

## Abstract

**Background:**

10 Hz electroencephalographic (EEG) alpha rhythms correlate with memory performance. Alpha and memory decline in older people. We wished to test if alpha-like EEG activity contributes to memory formation. Flicker can elicit alpha-like EEG activity. We tested if alpha-frequency flicker enhances memory in older people. Pariticpants aged 67–92 identified short words that followed 1 s of flicker at 9.0 Hz, 9.5 Hz, 10.0 Hz, 10.2 Hz, 10.5 Hz, 11.0 Hz, 11.5 Hz or 500 Hz. A few minutes later, we tested participants' recognition of the words (without flicker).

**Results:**

Flicker frequencies close to 10 Hz (9.5–11.0 Hz) facilitated the identification of the test words in older participants. The same flicker frequencies increased recognition of the words more than other frequencies (9.0 Hz, 11.5 Hz and 500 Hz), irrespective of age.

**Conclusion:**

The frequency-specificity of flicker's effects in our participants paralleled the power spectrum of EEG alpha in the general population. This indicates that alpha-like EEG activity may subserve memory processes. Flicker may be able to help memory problems in older people.

## Introduction

The principal electroencephalographic (EEG) rhythmic slow activity (RSA) – the 10–12 Hz alpha rhythm – relates to memory functions in healthy adults [1-7]. Alpha power may relate particularly to episodic memory [8,9]. It diminishes in old age [10] and in Alzheimer's disease [11-14], but anti-dementia drugs can increase it [15,16]. Earlier workers viewed EEG alpha rhythms as merely epiphenomenal [17,18]. More recent work has shown that EEG activity has a causal role in psychological functions [19-21], including memory [22,23]. This work is the basis of our study.

Animal studies provide experimental evidence that EEG rhythms may modulate memory. Rhythmic slow activity (RSA) in the hippocampus facilitates long-term potentiation (LTP) [24,25], the likely neural substrate of memory [26]. Modulating RSA using drugs can enhance memory [27,28]. Brain stimulation that elicits RSA can also enhance memory [22,23]. Moreover, the behavioural effects of RSA modulation are exquisitely frequency specific [21,29]. This frequency-specificity excludes the possibility that the stimulation alters behaviour non-specifically (e.g. by metal ion deposition). Taken together, the findings of drug and stimulation studies indicate that RSA can enhance memory.

To test if EEG rhythms can enhance memory in man, we need to modulate them experimentally. Flickering light induces frequency-locked EEG activity that can resemble endogenous alpha [30,31]. Flicker may even induce alpha-like activity [32]. We previously showed that alpha-frequency flicker could enhance memory [33]. Moreover, the flicker effects were highly frequency-specific: 10.0 Hz flicker enhanced recognition, but 8.7 Hz and 11.7 Hz were ineffective. This frequency-specificity makes it very unlikely that the flicker in our previous study modulated memory by non-specific mechanisms. Instead, our previous findings supported the view that alpha-frequency EEG activity may contribute to memory formation.

In older people, a fall of just 0.5 Hz in the endogenous alpha frequency with peak power relates to memory impairment [34]. We previously studied effects of flicker only at 8.7 Hz, 10.0 Hz or 11.7 Hz in young adults [33]. The first goal of the present study was to test the frequency-specificity of flicker's effects on memory at a higher resolution. If flicker alters memory by eliciting EEG alpha-like activity, then frequencies very close to the main EEG alpha frequency should enhance memory maximally. We tested this hypothesis by comparing flicker frequencies in 0.5 Hz steps around 10.2 Hz – the endogenous EEG alpha frequency with peak power [35]. Specifically, we tested if only flicker in the central range of endogenous alpha frequencies, close to 10.2 Hz, would enhance recognition, as in our previous study.

Our earlier study used a single low intensity of flicker at participants' fixation point. The present study used three intensities of flicker in the peripheral visual field. Higher intensities should elicit larger alpha-like EEG activity. Our second goal was to test if any memory-enhancing effects of peripheral flicker would show a "dose-response" relation with its intensity

Flicker can elicit large alpha-like EEG activity in healthy older people [36]. However, the amplitude and frequency of endogenous EEG alpha fall with age [35]. This fall is greater in those with mild memory problems [34]. This could have two possible corollaries for flicker's effect on memory. Age-related impairments in brain systems that subserve EEG activity might make older peoples' memories unresponsive to flicker. Conversely, if flicker-stimulated EEG activity can replace or restore endogenous alpha rhythms, then flicker's effects might be stronger in older people with poorer memories. Our final goal was to choose between these alternatives. To this end, we tested if flicker's effects depended on age.

In our study, flicker at 9.5–11.0 Hz (close to the frequency of alpha that shows peak power in the general population) facilitated the identification of short words during the initial 'learning phase' of the study. The same flicker frequencies enhanced the recognition of the words a few minutes later in the 'test phase'. Both the above effects were frequency-specific: control flicker frequencies (9.0 Hz 11.5 Hz, and 500 Hz) further from the central alpha frequency had no effects. These results support our hypothesis that alpha-like EEG rhythms contribute to memory in older people.

## Results

### Participant characteristics

The participants' median age was 78.5 years (range 67 to 92). 16 were men. The median MMSE score was 30 (range 28–30) and the median recall score in the HVLT was 29 (range 25 to 35).

### Learning phase

**Figure 1:**
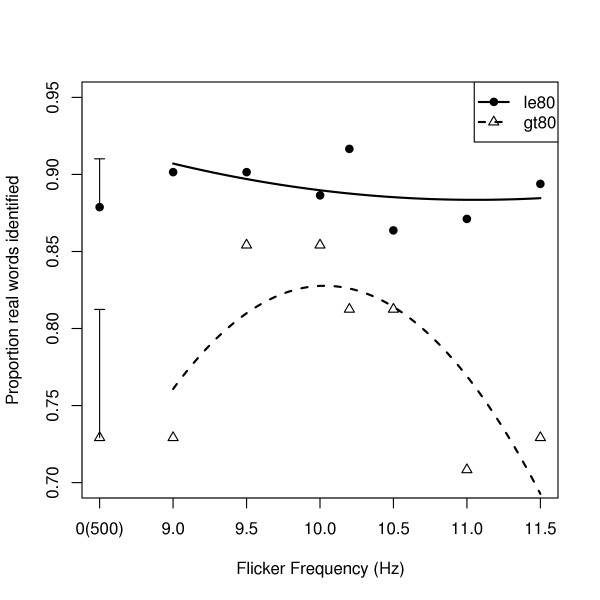
shows the proportion of real words that participants identified during the learning phase (ordinate) according to the frequency of flicker that immediately preceded them (abscissa). The points are the mean proportions of raw data, and the curves are the fitted quadratic trends over visible flicker frequencies. The solid circles and solid line are for participants aged 80 or less. The open triangles and dashed line are for participants older than 80 years. Note that flicker was not visible in the control (500 Hz) condition, so its effective frequency was 0 Hz. The error bars represent the overall standard errors of the means for each age group.

Overall, participants identified 90% of the 48 real-word trigrams in the learning phase (median correct = 90%, inter-quartile range 82–93%). The older participants (aged over 80) tended, not quite significantly, to identify fewer real words than the younger (t = -1.78, 1400 df, p = 0.085). However, flicker frequencies close to 10.2 Hz (9.5–10.5 Hz) restored the older participants' accuracy in identifying real words to that seen in the younger (Figure 1) (Quadratic trend of frequency-within-flicker × Age: t = -2.12, 1400 df, p = 0.03).

### Test phase

**Figure 2:**
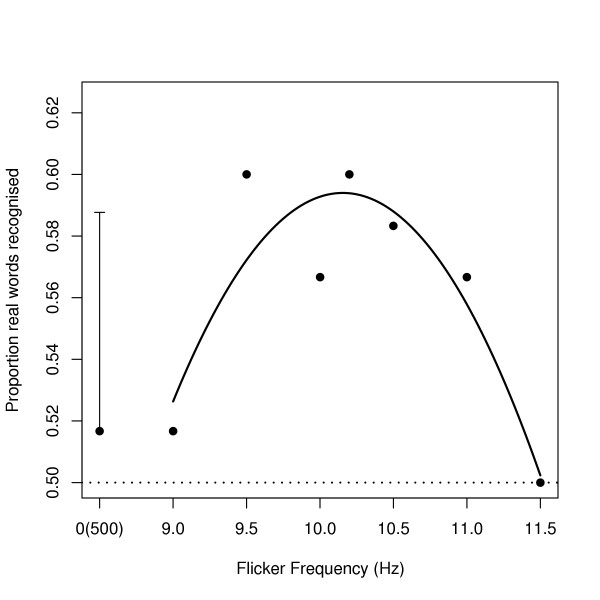
shows the proportion of words that participants recognised in the test phase (ordinate) according to the frequency of flicker that preceded them in the learning phase (abscissa). The points are the mean proportions of raw data for flicker at 0–150 mcd and the curve is the fitted quadratic trend over visible flicker frequencies from the analysis (note that flicker was not visible in the 500 Hz condition, so its effective frequency was 0 Hz). The error bar represents the overall standard error of the mean.

As we predicted, flicker frequencies close to 10.2 Hz increased later recognition of the real words from the learning phase (see Figure 2) (Quadratic trend of frequency-within-flicker: t = -2.13, 1400 df, p = 0.017, 1-tailed). Moreover, this effect showed an intensity-response relation (Linear trend of intensity × quadratic trend of frequency-within-flicker: t = -2.17, 1400 df, p = 0.015, 1-tailed).

Participants were less likely to recognize real words in the test phase that they had not identified during the learning phase. Hence, error rates in the two phases correlated (Spearman's ρ = 0.40, p = 0.03). We therefore tested if flicker's effects on recognition in the test phase were independent of its effects on identification in the learning phase. To this end, we covaried identification in the analysis of recognition. Even then, the quadratic trends over frequency-within-flicker remained significant (Quadratic trend of frequency-within-flicker: t = -1.79, 1399 df, p = 0.037, 1-tailed. Linear trend of intensity × quadratic trend of frequency-within-flicker: t = -1.69, 1399 df, p = 0.045, 1-tailed).

## Discussion

Flicker induces frequency-locked EEG activity over a wide range of frequencies, but maximally at the frequencies of the endogenous alpha rhythm (10 Hz) and its harmonics [31,37]. We presented flicker during memory encoding and found that only frequencies closer to 10.2 Hz –the endogenous alpha frequency with peak power – enhanced later recognition. Our study provides a stringent test of our *a priori *hypothesis that only flicker frequencies close to the peak frequency of endogenous alpha would enhance memory. Our control frequencies (9.0 Hz and 11.5 Hz) are within the range of EEG alpha, but endogenous alpha power at these frequencies is usually less than that at frequencies closer to 10.2 Hz [35]. Hence our results support the hypothesis (see Introduction) that flicker-induced alpha-like EEG activity selectively facilitates neural mechanisms of memory.

The present study did not record EEG responses. Our previous study showed stimulus- and frequency-locked EEG responses to flicker [33]. Even so, this does not prove that flicker altered recognition by modulating EEG activity directly, because flicker can elicit endogenous alpha [32]. The peak power of endogenous alpha varies within and between individuals [35]. Our unpublished analyses found important variation between individuals' responses to different flicker frequencies. Including this variation in the half-logit glmmPQL improved the model (reduced its Aikake Information Criterion by 4.3%) and slightly increased the significance of the fixed effects. However, even if alpha-frequency flicker enhanced memory by eliciting endogenous alpha, this would support our hypothesis that alpha-like EEG activity contributes to memory formation. Our results are thus consistent with previous work [38,39] indicating that flicker can probe the functions of EEG rhythms. Even if flicker's effects on memory were even more indirect (e.g. via changes in mood or arousal – see below), the frequency-specificity of flicker's effects – with effective frequencies centred on 10.2 Hz, the frequency of endogenous alpha with peak power – makes it hard to ascribe flicker's effects to mechanisms that do not relate to brain EEG-like activity. Therefore, our results also support the wider view (see Introduction) that EEG activity is not merely epiphenomenal, but can cause psychological states [19-23].

Our memory task was difficult and recognition was at chance levels in the control condition. This makes the flicker-induced enhancement of memory more striking. In effect, flicker close to 10.2 Hz caused recognition where there was none without it. We have previously shown that 10.0 Hz flicker enhanced recognition memory in young adults [33]. The present study supports and extends those previous findings by showing that flicker frequencies close to 10.2 Hz can enhance memory in cognitively-healthy older people. Moreover, earlier work [38,39] showed that gamma-frequency flicker elicited gamma-like activity and facilitated the perception of Kanisza figures. Taken together, the present and previous results support the view that flicker-evoked EEG activity can have functional effects paralleling those of endogenous EEG rhythms.

The frequency-specificity of flicker's effect on memory showed an intensity-response relation. This was consistent with our expectation, since more intense flicker should elicit larger EEG alpha-like activity. The present LEDs were brighter than the flicker in our previous study [33], where flicker was central. In the present study, the LEDs were in the peripheral visual field and many participants reported that they were unaware of them during the test. The fact that peripheral flicker can enhance memory means that it may be easier to use flicker for this purpose outside the laboratory.

Our recognition task tested episodic memory. The stimuli were all short words in common use and the task was to remember if they had occurred earlier in the paradigm. Our finding that flicker close to 10 Hz enhanced this episodic recognition memory parallels the observation that memory tasks which emphasise episodic memory elicit EEG alpha synchronization [8,9]. A plausible mechanism for this enhancement is that flicker-induced rhythmic EEG activity may increase "gain" within recurrent cortico-cortical and cortico-thalamic loops [40,41]. A second possibility is that the flicker-induced activity may facilitate long-term potentiation (LTP) in the hippocampus [24-26,42], which subserves episodic memory [43]. Our study cannot illuminate the neural mechanisms of filcker's effects. However, it can exclude the possibility that flicker altered recognition indirectly, via conditioning. This is because, if particular flicker frequencies had unconditioned reinforcing properties, then flicker should influence the recognition of words that precede it, not of those that follow it. We found no effect of flicker on memory for the preceding words (unpublished analyses). Previous work has shown that flicker does not influence subjective mood, but may influence alertness [44]. This may fit with findings that EEG rhythms relate to attentional switching in man [45] and rodents [46]. Hence, if flicker-elicited EEG activity simulates endogenous alpha, this could facilitate memory via attentional mechanisms.

The duration of flicker stimulation in each trial was only 1 s. The fact that such short-duration stimulation could enhance recognition may be consistent with evidence that endogenous alpha power synchronises only briefly during memory encoding [7]. On the other hand, the interval between flicker epochs was only 1 s and photic stimulation effects may persist over this time [32]. Further studies should test if the duration and timing of flicker alter its effects on memory. For now, we note that the efficacy of short-duration flicker has a practical corollary. It makes it easier to study flicker's effects using within-participant designs to control stringently for non-EEG effects of flicker, as here.

Flicker frequencies close to 10.2 Hz enhanced the identification of real words in the learning phase *only *in older participants (aged over 80). Consistent with our previous report [33], flicker did not alter the identification of real words during the learning phase in younger participants. A likely mechanism of the enhanced identification that we found here is that flicker simply accelerated responding in older participants, since they presumably knew the stimulus words as well as the younger ones. Such simple acceleration could apparently restore older participants' accuracy. Whatever its mechanism, this observation further supports the view that flicker frequencies near 10 Hz can enhance psychological processing. It also indicates that old age may amplify, rather than prevent, flicker's effects. The enhancement of memory by flicker frequencies close to 10.2 Hz was independent of performance in the learning phase. It was also independent of age. It is tempting, therefore, to speculate that 10 Hz flicker may help memory problems in older people. Cholinesterase inhibitors facilitate EEG alpha rhythms [15,16]. Our results suggest that this facilitation may underlie their anti-dementia effects. If so, our present results raise the possibility that flicker at frequencies close to 10 Hz could supplement or supplant the effects of cholinesterase inhibitors in patients with early dementia.

## Participants and methods

### Participants

All procedures complied with the Helsinki Declaraion and received prior approval from the Central Oxford Research Ethics Committee (COREC #1656). We recruited 30 cognitively healthy participants from the Foresight-Challenge cohort (see [47]). Exclusion criteria were a history of epilepsy or head injury (to minimise any risk of photosensitive seizures – [48]). Participants underwent the Mini Mental State Examination (MMSE – [49]) and Hopkins verbal learning test (HVLT – [50]) a year before the present study.

### Test paradigm

Tests used a desktop computer in a windowless room with fluorescent lighting. The computer screen displayed test items near its centre, in white letters 2.5 cm high. 18 red (wavelength = 625 nm) light-emitting diodes (LEDs) (Kingbright L-813ID, part number 179-026 Farnell InOne, Canal Road, Leeds LS12 2TU), in two 3 × 3 arrays measuring 3.3 × 3.3 cm, stood on top of the screen. Each LED had a maximum luminance of 150 millicandelas, and emitted maximum light of 0.72 Lux. A flicker generator could drive the LEDs to flicker at 9.0 Hz, 9.5 Hz, 10.0 Hz, 10.2 Hz, 10.5 Hz, 11.0 Hz, 11.5 Hz and 500 Hz (no discernible flicker). The flicker's waveform was triangular. The computer programmed flicker at these eight frequencies and at three intensities (maximal light = 0.24, 0.48 and 0.72 Lux), in a balanced design.

The test items were three-letter character strings (trigrams) in consonant-vowel-consonant format. We created two pools of about 400 trigrams. The first consisted of 3-letter words in common use. This pool excluded salient trigrams (e.g. SEX) and uncommon words (e.g. FEZ, which we used for practice items). The second pool consisted of about 400 nonsense trigrams (e.g. GEC). For each participant, the computer drew 96 trigrams, without replacement, from the pool of real common words and 48 trigrams from the nonsense pool.

The task had three phases: practice, learning and test. There were 10 trials in the practice, 48 in the learning, and 48 in the test phases. Each trial lasted 2000 ms. For the first 250 ms, the computer screen was blank. Then, a pair of fixation crosses appeared for 250 ms, 3 cm on either side of the screen's centre. Finally, a pair of trigrams replaced the fixation crosses. The screen displayed the trigrams in capital letters 1.5 cm high and about 1.2 cm wide: the middle letters of each trigram occupied the locations of the fixation crosses. The trigrams remained on the screen for 1500 ms, until the beginning of the next trial. Trials in the learning phase began with 1000 ms of flicker, when the screen was blank. This continued for 500 ms before and 500 ms after the trigrams appeared. Participants held the computer mouse in both hands with a thumb on each of its buttons. They could respond by pressing one of the buttons. The computer only recorded responses made between 250 ms after the appearance of the trigrams and the beginning of the next trial.

### Test procedure

All participants began with a practice run that presented 10 pairs of trigrams. In each pair, one trigram was a real word and the other was a nonsense combination. We asked participants to identify the real word and press the button on that side. Participants could only progress to the learning phase if they achieved 80% correct responses during the practice phase. If participants did not achieve 80% immediately, we recycled the practice stimuli a maximum of 3 times. The practice used 10 uncommon real 3-letter words (e.g. FEZ) that did not occur in the test, later.

The learning phase gave participants the opportunity to learn 48 real-word trigrams. It presented 48 pairs of trigrams, each with one real and one nonsense word. The program randomly assigned the real word in each pair to appear the right or left side of the screen. We asked participants to identify the real word and click the button on the same side. We told them that we would test their memory for the real words later. A 1000 ms period of flicker preceded the appearance of each pair of trigrams by 500 ms (see above). The computer varied the frequency and intensity of the flicker in a quasi-random, but balanced, design that was unique for each participant. Hence, 6 pairs of trigrams followed presentation of flicker at each frequency, with 2 at each frequency-intensity combination.

The test phase followed about 2 minutes after the learning phase. There was no flicker during the test. The computer presented 48 pairs of real words. One word of each pair was 'old', from the learning phase, and the other was 'new' in the test. Any real-word trigram could be old or new, for different participants. The program randomly assigned the position of the old word to right or left. The participants' task was to choose the old word they had seen in the test phase by pressing the mouse button on that side. The computer counted correct responses to the old trigrams, according to the flicker frequency that had preceded each during the learning stage.

### Statistics

All analyses used the R programming language [51]. We analysed our repeated-measures binary response data using generalised linear mixed modeling via penalised quasi likelihood (glmmPQL) [52] to estimate flicker frequency effects within participants. Our task had a forced-choice response format, so participants must score at chance (50%) if they cannot identify or recognise the target words. Therefore, our glmmPQL used a half-logit transformation of the logistic [1/2 + 1/2(logit)] as its link function [53]. We used half-logit glmmPQL to analyse the identification of real words in the learning phase, and the recognition of the same real words in the test phase.

The half-logit regressions centred age at 80 years and included interaction terms of flicker with age and intensity. They first fitted a term to test for frequency-non-specific effects of flicker by comparing trials with no discernible flicker (500 Hz) to all those with flicker in the alpha range (9.0–11.5 Hz, combined). This term tests whether discernible flicker has overall effects that are not frequency-specific. The design then nested orthogonal terms coding linear and quadratic trends over flicker frequency within the non-specific flicker term. The quadratic trend stringently tests the hypothesis that only flicker frequencies close to 10.2 Hz would enhance memory.

To test if higher LED intensity would increase frequency-specific flicker effects, we included the interactions of the linear and quadratic trends over flicker frequency with the LED intensity. To test if flicker's effects vary with participants' age, we included the interactions of the polynomial trends over flicker frequency with age. The analyses included random intercepts and accounted for the temporal autocorrelation of errors over trials by modeling the error matrix with an auto-correlation structure of order 1 [54].

## Authors' contributions

JHW conceived the study and wrote the Pascal program to perform it. DR recruited participants and guided them through the procedure. AO wrote the half-logit link function for the glmmPQL procedure. JW and DR wrote the manuscript. All authors read and approved the final manuscript.
